# Integration of cellular-resolution optical coherence tomography and Raman spectroscopy for discrimination of skin cancer cells with machine learning

**DOI:** 10.1117/1.JBO.28.9.096005

**Published:** 2023-09-14

**Authors:** Cian You, Jui-Yun Yi, Ting-Wei Hsu, Sheng-Lung Huang

**Affiliations:** a National Taiwan University, Graduate Institute of Photonics and Optoelectronics, Taipei, Taiwan; b National Kaohsiung Normal University, Department of Electrical Engineering, Kaohsiung, Taiwan; c National Taiwan University, All Vista Healthcare Center, Taipei, Taiwan

**Keywords:** optical coherence tomography, Raman spectroscopy, skin cancer cells, machine learning

## Abstract

**Significance:**

An integrated cellular-resolution optical coherence tomography (OCT) module with near-infrared Raman spectroscopy was developed on the discrimination of various skin cancer cells and normal cells. Micron-level three-dimensional (3D) spatial resolution and the spectroscopic capability on chemical component determination can be obtained simultaneously.

**Aim:**

We experimentally verified the effectiveness of morphology, intensity, and spectroscopy features for discriminating skin cells.

**Approach:**

Both spatial and spectroscopic features were employed for the discrimination of five types of skin cells, including keratinocytes (HaCaT), the cell line of squamous cell carcinoma (A431), the cell line of basal cell carcinoma (BCC-1/KMC), primary melanocytes, and the cell line of melanoma (A375). The cell volume, compactness, surface roughness, average intensity, and internal intensity standard deviation were extracted from the 3D OCT images. After removing the fluorescence components from the acquired Raman spectra, the entire spectra (600 to $2100\;{\mathrm{cm}}^{-1}$) were used.

**Results:**

An accuracy of 85% in classifying five types of skin cells was achieved. The cellular-resolution OCT images effectively differentiate cancer and normal cells, whereas Raman spectroscopy can distinguish the cancer cells with nearly 100% accuracy.

**Conclusions:**

Among the OCT image features, cell surface roughness, internal average intensity, and standard deviation of internal intensity distribution effectively differentiate the cancerous and normal cells. The three features also worked well in sorting the keratinocyte and melanocyte. Using the full Raman spectra, the melanoma and keratinocyte-based cell carcinoma cancer cells can be discriminated effectively.

## 1 Introduction

Skin cancer is the most common cancer category and is among the most costly of all cancers to treat.^1^ One in every three cancers is diagnosed as skin cancer. The most common skin cancer, which belongs to the development of keratinocyte cells, is basal cell carcinoma (BCC) and squamous cell carcinoma (SCC). The third most common one is melanoma, and these three skin cancers account for ∼99% of skin cancers. The incidence of skin cancer is increasing year by year.^2^^–^^5^ BCC and SCC are treatable, especially in the early stages of cancer.^6^ Although their mortality rate remains low,^7^^,^^8^ the incidence of both in recent years continues to rise. According to melanoma statistics in 2023,^9^ if the cancer is found and excised in situ, the patient’s survival rate is >99% on average. However, if the cancerous tumor metastasizes, the patient’s 5-year survival rate will be reduced to 32%. Therefore, if it can be found early, the survival rate of patients can be significantly improved. Also, it was indicated that the skin cell lines could be effective in cancer models,^10^ and the cancerous cell has an apparent protrusion.^11^ Therefore, microscopic measurement of skin cells can be performed by optical techniques, and it can be used as a model for pre-cancerous skin cell lesions. It is expected to be used as a basis for pre-cancerous judgment, bringing significant benefits to detection, prevention, and treatment and saving medical costs.

OCT has the advantages of non-invasiveness, real-time, sub-micron resolution, easy sample fabrication, and no need for dye calibration on single-cell and tissue-level imaging.^12^^,^^13^ Other imaging techniques for measuring single cells include cryo-electron tomography,^14^ optical projection tomography,^15^^,^^16^ x-ray tomography,^17^ magnetic resonance imaging,^18^ multiphoton microscopy,^19^ and so on. However, these techniques have not been used clinically for *in vivo* cellular resolution imaging.

Full-field optical coherence tomography (FF-OCT) is a variant of OCT, which also have high-resolution and non-invasive scanning advantages.^20^^,^^21^ It captures the en-face image at high speed using charge-coupled devices or complementary metal-oxide semiconductor cameras. In addition, FF-OCT can provide near-isotropic axial and lateral resolutions for accurate cell imaging. The three-dimensional (3D) microstructure of skin cells may change when lesions occur.^11^ With our cell-level and isotropic resolution FF-OCT system, various 3D features, such as volume, compactness, surface roughness, average intensity, and internal intensity standard deviation of cells, were obtained to differentiate in morphology between cancer cells and normal ones. Cell compactness was used to quantify the tumor shape of cervical cancer.^22^ The compactness of a cell itself can be regarded as one of the critical indicators of regenerative medicine.^23^ When the shape is closer to a circle or a sphere, the higher the compactness, and the compactness value will be closer to 1. Surface roughness is defined as the square root of the surface contour minus the surface average. It can be used to describe the degree of surface undulation. In previous studies, the cellular morphology change and carcinogenesis can be detected by the average intensity of the cells.^11^^,^^24^^,^^25^ In this study, we compare these 3D parameters with cancer cells and normal ones to distinguish whether the cells are cancerous or not. The skin cells in this experiment are mainly divided into two categories: the first one is the keratinocyte-based cells, including the cell line of keratinocytes (HaCaT), SCC (A431), and BCC (BCC-1/KMC; abbreviated as BCC), and the second one is melanocyte-based cells, including primary melanin cells and melanoma cell lines (A375).

It should be noted that quantizing the morphology and intensity distribution of skin cells has not been able to distinguish cells perfectly, especially between cancer cells. Thus, it is difficult to differentiate by OCT alone.^11^ The intracellular chemical bonding components of skin cancer cells are quite differentiative^26^ and can be detected by Raman spectroscopies (RSs) for auxiliary judgment. Its principle is to detect the spectrum of inelastically scattered light generated by the internal molecular bond’s vibration modes when the pump light incidents into the sample. RSs have been used to study the classification of label-free cancer cells and tissues.^27^^–^^29^ With the integration of OCT and RS, the 3D morphological details and chemical compositions can be acquired simultaneously.

Combined RS-OCT systems have already been developed and utilized for the classification of abdominal tissues classification,^30^ and skin and lung tumors.^31^ Moreover, the RS-OCT system was made a clinical instrument capable of both morphological and biochemical characterization of skin cancers.^32^ However, submicron-resolution OCT has not been used so far to differentiate the cellular features. With machine learning algorithms, this work expects that the classification between cancer cells and normal cells can be accurate and beneficial to clinical applications.

## 2 Methods

### 2.1 Instrumentation

**Fig. 1:**
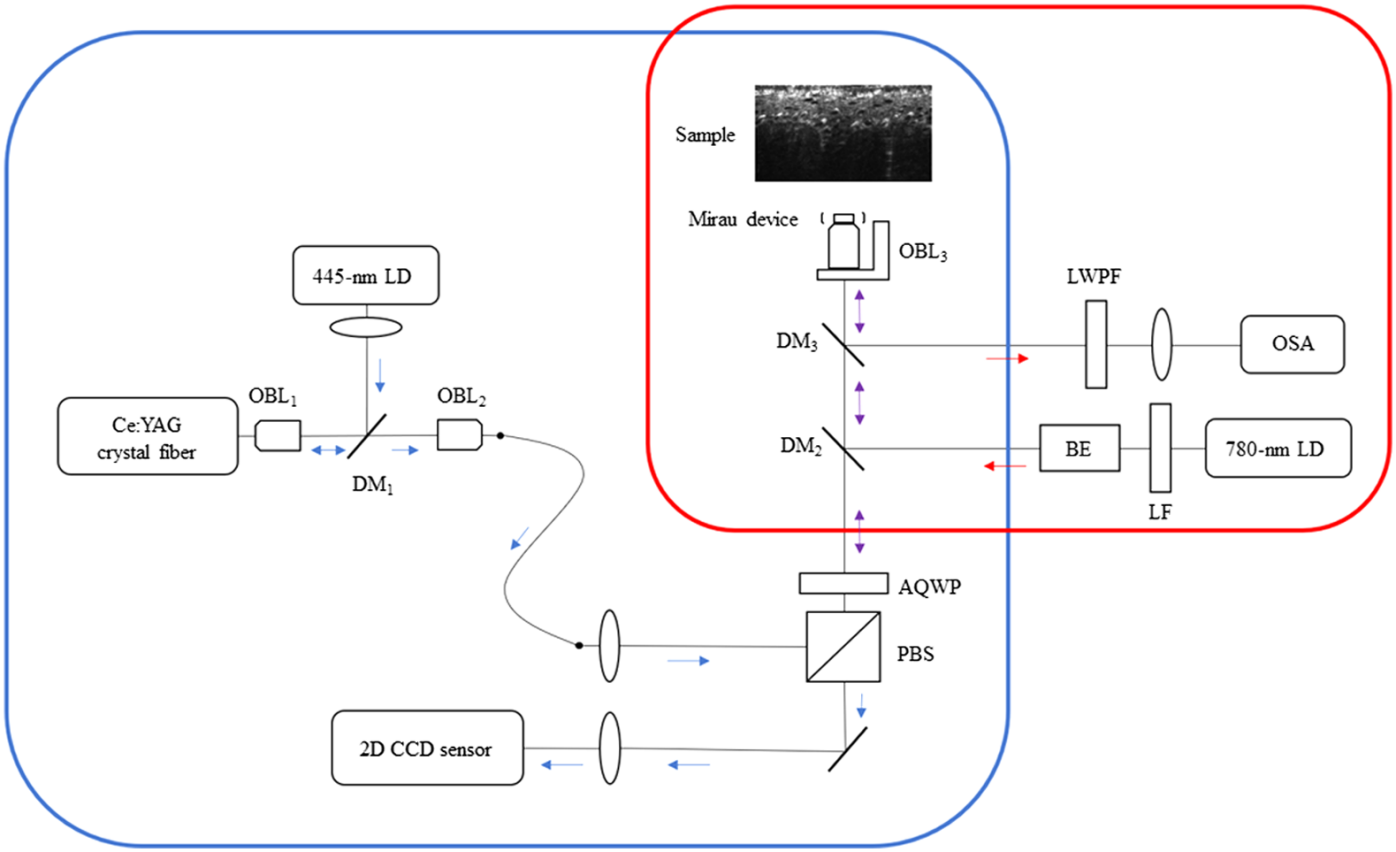
Schematic of the integrated OCT (blue box) and Raman (red box) system. The OCT comprises the Ce:YAG crystal fiber light source, a Mirau-type interferometer, and a 2D CCD sensor for fast image acquisition. The Raman system includes an NIR narrow-band laser, a long-wavelength-pass filter, and an OSA. The optics in the Mirau module has AR coatings around the 780 nm wavelength. OBL, objective lens; PBS, polarizing beam splitter; AQWP, achromatic quarter-wave plate; DM, dichroic mirror; LF, line filter; BE, beam expander; LWPF, long-wavelength-pass filter.

An integrated OCT and Raman system was built to acquire the 3D structure of skin cells and their chemical compositions. As shown in Fig. 1, the sample arms of the OCT and Raman modules are kept in the same optical path so the two beams can fall in the same lateral position.

**Fig. 2:**
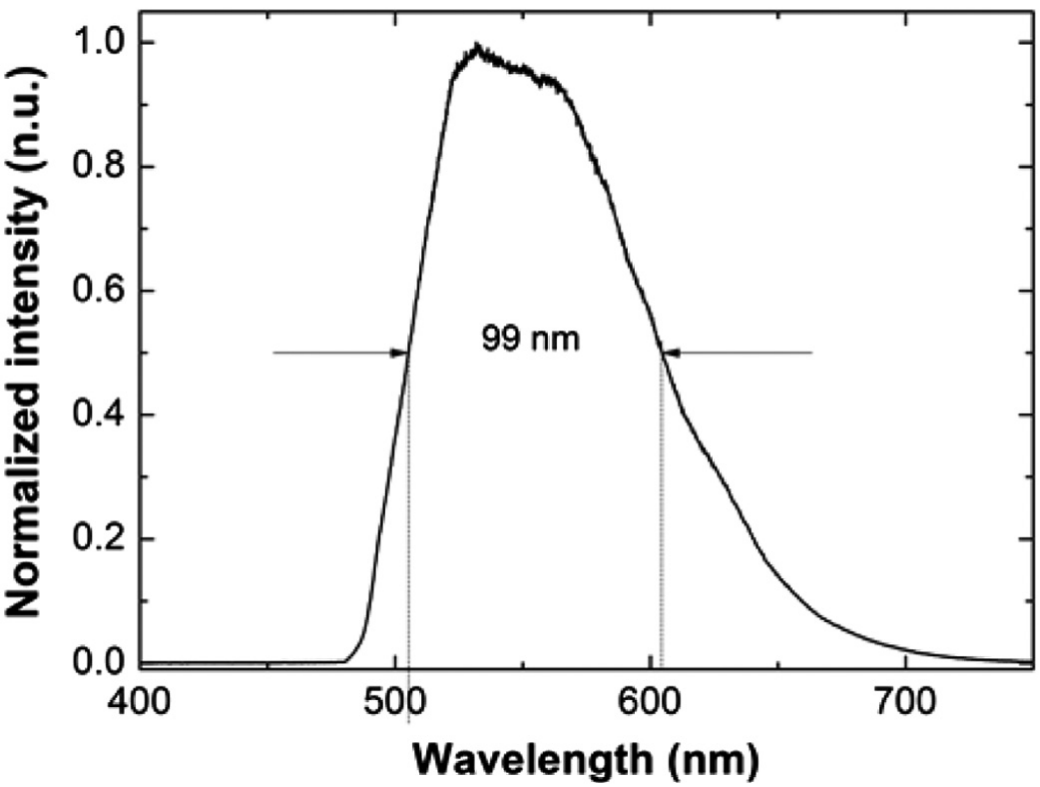
Output spectrum of ${\mathrm{Ce}}^{3+}:\mathrm{YAG}$ crystal fiber.

The OCT’s light source uses a 445-nm blue laser diode as the pumping source, which is collimated by an aspheric lens and then reflected into a 40× objective lens (PLN 40X, Olympus) by a dichroic mirror (LM01-466-25, Semrock) to focus the laser on the crystal fiber for the broadband light source generation. The crystal fiber with silver plating at one end was operated in a double-pass scheme. The residual laser light was filtered through the dichroic mirror to obtain a broadband light source having a center wavelength of 560 nm and a full width at half maximum of 99 nm, as shown in Fig. 2. The broadband emission was coupled into a multimode fiber through a 20× objective (RMS20X, Olympus). A Mirau-based FF-OCT was employed as the backbone for Raman integration. The infinite-conjugate imaging system consists of a water immersion microscope objective (Umplanfl 20X/0.5, Olympus), a 45-deg mirror, a projection lens (AC254-150-A-ML, Thorlabs), and a two-dimensional (2D) CCD sensor (B0620M, IMPERX). The OCT has a penetration depth of nearly 200  μm. The total power on the cell line was 3 mW, which falls in the IEC Class 3R range and is not hazardous for the skin. The lateral and axial resolutions of OCT are 0.8 and 0.75  μm (in tissue), respectively.

The Raman module light source was a 780-nm LD with a volume holographic grating to reduce the line width to $10^{-3}$ to $10^{-4}\;\mathrm{nm}$. The near-infrared excitation has negligible fluorescence and a more than 400-μm penetration depth. The spectral side lobes of the LD were eliminated by a laser line filter (LL01-780-12.5, Semrock). The beam then enters a beam expander system consisting of a lens pair with focal lengths of 35 mm (AC254-35-B-ML, Thorlabs) and 300 mm (AC254-300-B-ML, Thorlabs). The second dichroic mirror, ${\mathrm{DM}}_{2}$ (DMSP650R, Thorlabs), reflects the light into the Mirau objective. The Raman excitation beam is then focused to 1.7  μm on the sample with a power of 10.5 mW and an optical slice thickness of 5.6  μm. In the Raman detection arm, the backscattered light from the sample is reflected by the third dichroic mirror, ${\mathrm{DM}}_{3}$ (DMSP805R, Thorlabs), followed by a long wavelength pass filter (LP02-785RS-25, Semrock) to block the residual excitation light and Rayleigh scattered light. Finally, the backscattered light is coupled into a fiber-coupled optical spectrum analyzer (WP785, Wasatch Photonics), which has a spectral resolution of $6\;{\mathrm{cm}}^{-1}$ at a wavelength range of 802 to 932 nm (350 to $2100\;{\mathrm{cm}}^{-1}$). The mechanical stage of OCT scanning is also used here, but the distance of each movement is only one-quarter of the field of view (FOV) of the OCT along the x direction (72.9  μm) and one-third along the y direction (73.2  μm). Each sample will sweep 12 points with an integration time of 4 s and an average of 100 measurements. It should be noted that the laser power of the Raman system is 10.5 mW at 780 nm, which falls in IEC Class 3B, and may be hazardous for the skin. In our experiments, no visible damage was observed after the measurements.

### 2.2 Sample Preparation

The cell lines were cultured with DMEM-high glucose (GibcoTM) and 10% fetal bovine serum solution in a petri dish, then placed in an incubator at a constant 37°C and 5% carbon dioxide concentration to proliferate. After about 1 week of incubation, trypsin-EDTA is added to the dish to decompose the attached proteins between the cells and the culture dish. When the cells are detached from the culture dish, an appropriate serum is added to terminate the trypsin action. And the cell preparation was completed by adding 4% of a fixed solution (paraformaldehyde) to the culture dish. Previous studies have shown that using formalin for fixation affects cells’ lipids and protein components.^33^^,^^34^ The 4% paraformaldehyde solution causes changes in the cells’ Raman spectrum, but the effect is smaller than that of other solutions.^35^ A cell concentration of $4\times 10^{7}\text{ cells}/\mu l$ was used, and there was no agarose as the cell base material in the Raman measurement. The cell line soaked in 4% fixative (trioxane) was directly aspirated by a burette and dropped on fused silica to complete the sample preparation.

### 2.3 OCT Imaging and Raman Spectroscopy Protocol

**Fig. 3:**
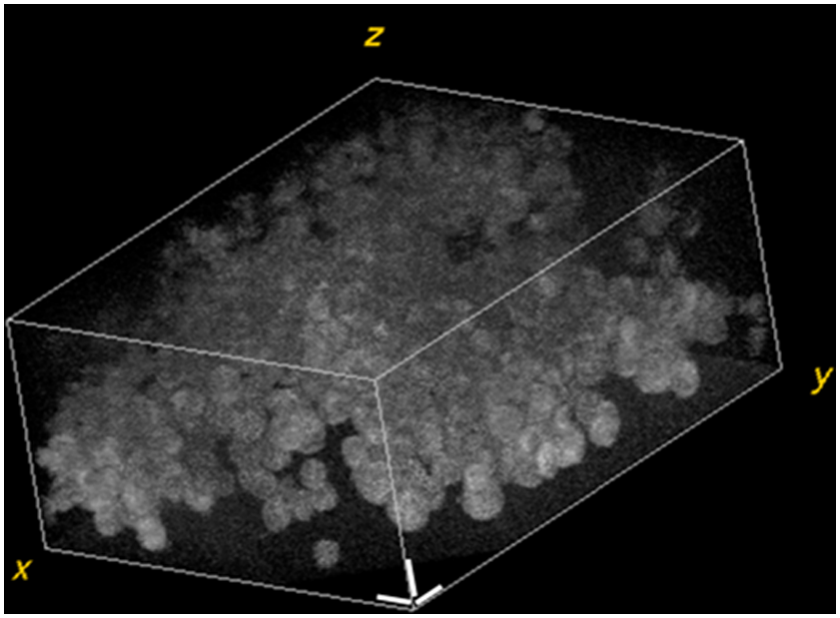
A 3D OCT image of the melanoma cell line. The scale bar is 20  μm.

The OCT scans the skin cells with a 2D translational stage to expand the FOV, and the images were stitched to form a larger 3D volume. A z-axis scan is performed for one FOV each time, then moved horizontally to the next FOV, and the same z-axis scan is completed. Finally, the 3D stitched volume is about 1166.4  μm×878.4  μm×200  μm (z-direction), composed of 16 pieces of FOV (4 by 4). The z-axis scan speed was 0.812  μm/s, so it took about 3 min to scan a 3D volume of a single FOV. The 3D volumetric data of one FOV (291.6  μm×219.6  μm×200  μm), as shown in Fig. 3, was analyzed quantitatively by the ImageJ^®^, which is an open-source image processing and analysis program to segment the cells and calculate various 3D features.

The image processing has four steps: pre-processing, Gaussian filtering, binarization operation, and measurement analysis. The 3D image is chopped in the pre-processing to remove the strong reflection interface and the bottom cell-free signal. The second step is to perform Gaussian filtering, which results in the convolution of the original image with the Gaussian functions shown in Eqs. (1) and (2). One is to perform Gaussian filtering in the horizontal plane (i.e., x-y plane), and another is to perform Gaussian filtering in the depth direction (z-axis):

$$G(r)=\frac{1}{2\pi \sigma_{r}^{2}}e^{-\frac{r^{2}}{2\sigma_{r}^{2}}},$$ (1)

$$G(z)=\frac{1}{\sqrt{2\pi }\sigma_{z}}e^{-\frac{z^{2}}{2\sigma_{z}^{2}}},$$ (2)

where r is the filter radius ($r^{2}=x^{2}+y^{2}$). The σ values are selected to match the spatial resolutions in the x, y, and z directions. The pixel separations of our FF-OCT system are 0.45  μm in the x-y plane and 0.2  μm in the vertical direction (i.e., depth direction). We selected two pixels in the horizontal directions (i.e., x-y plane) and 4.5 pixels in the vertical direction (i.e., the depth direction) to match our system resolution.

The third step is the binarization process, which can be subdivided into threshold setting, watershed, and erosion operations. The threshold setting is based on ImageJ^®^’s built-in auto threshold function to differentiate the inter-cell and extra-cell regions.^36^^,^^37^ The watershed algorithm segments the original cell clusters and the interference of cell debris. The structural element during the erosion process was 3 pixels by 3 pixels. After the mean threshold method, the image is slightly expanded, so erosion is used to retain cell images. Finally, skin cell capture and feature quantification were performed using 3D object counter and 3D ROI manager in ImageJ^®^.

### 2.4 Machine Learning Protocol

The cell numbers of keratinocytes (HaCaT), SCC (A431), BCC (BCC-1/KMC), primary melanocyte cells, and melanoma cancer cell lines (A375) are 119, 58, 74, 36, and 227, respectively. The cell volume, compactness, surface roughness, average intensity, and internal intensity standard deviation were extracted from the OCT images.^38^ After removing the fluorescence components from the acquired Raman spectra, the entire spectra (600 to $2100\;{\mathrm{cm}}^{-1}$) were used. Three discriminating algorithms, i.e., linear discriminant analysis (LDA),^39^ k-nearest neighbors (KNN) algorithm,^40^ and decision tree (TREE),^41^ were utilized with three common architectures for ensemble learning, i.e., bagging, boosting, and subspace.^42^

MATLAB was utilized for scripting the machine-learning algorithms with 10-fold cross-validation. About 10% data were used as the test set for each fold. The parameters of the LDA, KNN, and TREE are briefly described as follows:

#### 2.4.1 Linear discriminant analysis

Set the discriminant mode to linear (program: DiscrimType = ‘linear’); make the two parameters of the redundant value Delta and Gamma, and select the two parameters with the lowest error rate at the fewest observation data. Initial tests were performed. Since the number of samples is small but representative, they are all set to 0 (program: Delta = 0, Gamma = 0).

#### 2.4.2 k-nearest neighbors

Set the distance measurement method to Euclidean distance (program: Distance = ‘euclidean’); set the distance weight to be equal (program: DistanceWeight = ‘equal’); use the nearest neighbor number to 1 when sorting (program: NumNeighbors = 1). Finally, the standardization settings were not used to reduce the impact of outliers (program: Standardize = ‘false’).

#### 2.4.3 Decision tree

The maximum number of branches is set to the total number of samples minus one (program: MaxNumSplits = size(X, 1) - 1); the minimum number of leaves for a single branch is set to 1 (program: MinLeafSize = 1). Due to two class distinctions, the Gini coefficient was used as the classification criteria (program: SplitCriterion = ‘gdi’). Finally, we conducted classification testing with randomly selected measurements, where all the measured values of the samples were selected for classification to increase accuracy (program: NumVariablesToSample = ‘all’).

## 3 Results and Discussion

### 3.1 OCT Results

The skin cells of this experiment are mainly divided into two categories: the first is the keratinocyte-based cells, including the cell line of keratinocytes (HaCaT), the cell line of SCC (A431), and the cell line of BCC (BCC-1/KMC). The second category is the melanocyte-based cells, which contain primary melanin cells and melanoma cancer cell lines (A375).

#### 3.1.1 Images of keratinocyte-based cells

Keratinocytes are the most abundant cells in the epidermis. Both SCC and BCC are developed from keratinocytes. This study employs keratinocytes and cell lines of SCC and BCC. We used HaCaT (keratinocyte), A431 (SCC), and BCC-1/KMC (BCC) to analyze the 3D morphology and intensity of the original OCT images. 3D features were extracted from the isotropic and cellular-resolution OCT images, including volume, compactness, surface roughness, average intensity, and internal intensity standard deviation of cells. The “intensity” related features are average intensity and internal intensity standard deviation of cells. The “morphology” related features are volume, compactness, and surface roughness.

**Fig. 4:**
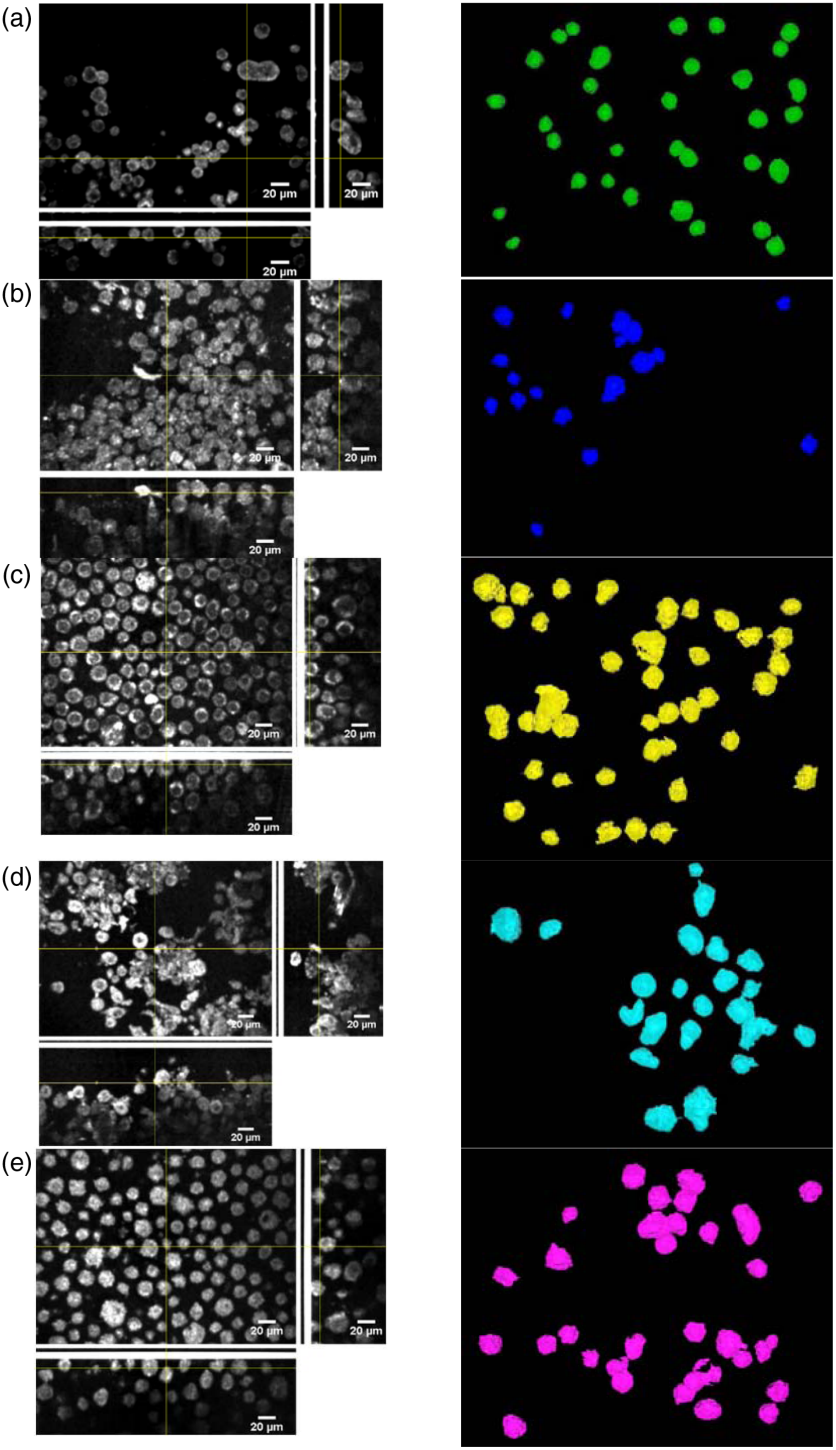
3D views (left column) and ImageJ processed images (right column) of (a) HaCaT, (b) A431, (c) BCC-1/KMC, (d) melanocyte, and (e) A375. The thick vertical and horizontal white lines in (a), (c), (d), and (e) of the left column represent the reflections from the glass slide in the Mirau module. The ImageJ delineates the boundary of the cells.

As shown in Fig. 4, HaCaT has a smooth surface, the cell shape is close to a circle in two dimensions, and the 3D shape approximates the spherical shape. On the contrary, cancer cells have a protrusion on the surface, which may be related to the aggressiveness of cancer cells.^11^^,^^24^ Cancer cells tend to grow out of a tentacle-like shape to expand their cancerous areas. In terms of brightness, the HaCaT cell line generally has the lowest average intensity, but it depicts the most significant internal contrast. The low average intensity may be due to the relative homogeneity of keratinocyte structure, so the backscattering light intensity drops significantly.^43^ Cancer cells are more irregular in appearance, so the backscattered light becomes more intense. Because of the above two characteristics, the machine-learning-based algorithm could distinguish cancer cells from normal cells.

#### 3.1.2 Images of melanocyte-based cells

Melanocytes are responsible for the production of melanin, which provides color to the skin. In this study, melanocyte and its cancer cell line, A375, were employed. Figures 4(d) and 4(e) show the OCT images of normal primary melanocyte cells and A375, respectively. Similarly, the cancer cell line (A375) has a large surface protrusion, and the cancer cells have a rough surface, which is related to the malignancy of cells.^24^ In addition, a small amount of melanin is in the cells, and melanin has a high complex refractive index.^44^ Therefore, the intensity of both cells is higher than other cell types.

#### 3.1.3 3D features analysis of skin cells

**Fig. 5:**
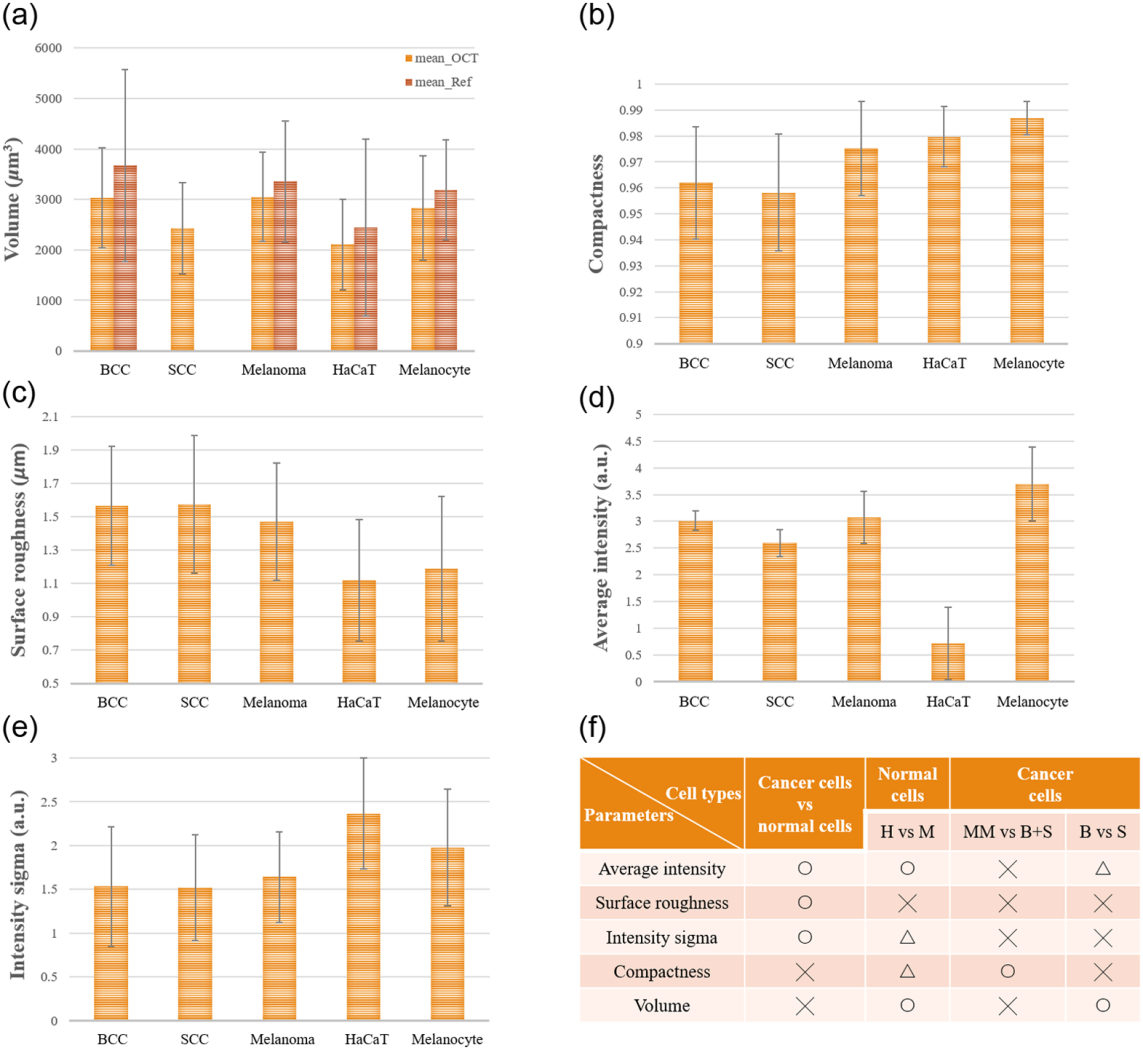
(a) Chart of skin cells’ volume compared with literature.^40^ Charts of skin cells’ comparison on (b) compactness, (c) surface roughness, (d) average intensity, and (e) intensity standard deviation. (f) Summary of the effectiveness of the five features on the classification of five cells (H, HaCaT; M, melanocyte; MM, melanoma; B, BCC; S, SCC; O, △, and X represent significant difference (p<0.001), slight difference (p<0.05), and almost no difference (p>0.05), respectively.

Figure 5 summarizes the features extracted from the five types of skin cells. As shown in Fig. 5(a), our experiment’s volume mean and standard deviation are generally consistent with that from the literature.^38^ The HaCaT cell line and the A431 cell line were cultured from the spinous layer cells and the granular layer, whereas the BCC cells were mainly cultured from the basal layer cells. The volume of BCC cells is larger than that of the spinous layer and the granular layer, which may be caused by different growth cycles and growth positions. The melanocyte-based cells all evolved from melanocytes, so there is no apparent volume variation. It is shown Fig. 5(b) that the normal cells (HaCaT and melanocyte) have slightly higher compactness than the cancer cells (BCC, SCC, and melanoma). It could be reflected that the formation of cancer cells in the form of protrusion,^11^^,^^24^ resulting in a shape that is not spherical. Compactness makes it possible to distinguish whether it is a cancer cell. The degree of cell surface roughness in cytology can reflect that it may be cancer cells.^11^^,^^24^ From Fig. 5(c), the surface roughness of cancer cells can be found to be much higher than that of normal cells. Therefore, surface roughness makes it possible to distinguish between normal and cancer cells.

In Fig. 5(d), it can be seen that the cells with a high gray value of keratinocyte-based cells are all cancerous cells. In contrast, the melanocyte-based cells exhibit an opposite trend, i.e., normal cells have a higher gray value than cancerous cells. The cytoplasm contains several micron-level organelles, such as the endoplasmic reticulum and mitochondria. The endoplasmic reticulum and peptide are layered, and the thickness of the single layer is less than the system resolution. But the mitochondria are ellipsoids, and the diameter^45^ is close to the system resolution. The long axis is slightly larger than the resolution, and their number is significant. It is speculated that the backscattering of the mitochondria mainly contributes to the scattered signals detected in the cells.^46^ In recent years, molecular biology studies have also observed variations in the mitochondria of normal cells and cancer cells, and mitochondria are the prominent organelles that provide energy for the cell. Therefore, when the cells are more active, the number of mitochondria may be higher, and there may be a clustering phenomenon. Thus, cancer cells can be detected with the results of the keratinocyte-based cell in Fig. 5(d) and the OCT image of Fig. 4. They have more strong backscattered light, so the overall average intensity of cancer cells is relatively high. However, the melanocyte-based cell is just the opposite. Although there are also differences in mitochondria, the high absorption rate and high refractive index of melanin^44^ have more influence than the former, so the difference in average intensity is mainly due to the amount of melanin. The reason is that primary melanin cells are closer to melanocytes in the living body, so the average cell intensity is slightly higher than melanoma cell lines cultured in multiple generations.

The cells’ internal intensity standard deviation can show the intracellular intensity distribution. From Fig. 5(e), it can be found that normal cells generally have a higher tendency. The previous OCT diagram shows that the nucleus and cytoplasm of normal cells are significantly different in intensity from cancer cells, so the classification of normal cells and cancer cells can clearly distinguish the differences. Therefore, this feature is also used as one of the parameters for determining cell types. As shown in Fig. 5(f), the average gray value, surface roughness, and internal intensity standard deviation can distinguish cancer cells from normal cells. In addition, the normal cells, i.e., HaCaT and melanocyte, can be differentiated by the average intensity feature. However, due to the significant standard deviation between cancer cells’ volume and compactness, the OCT image features may not effectively discriminate among the cancer cells.

### 3.2 Analysis of Raman Spectra

**Fig. 6:**
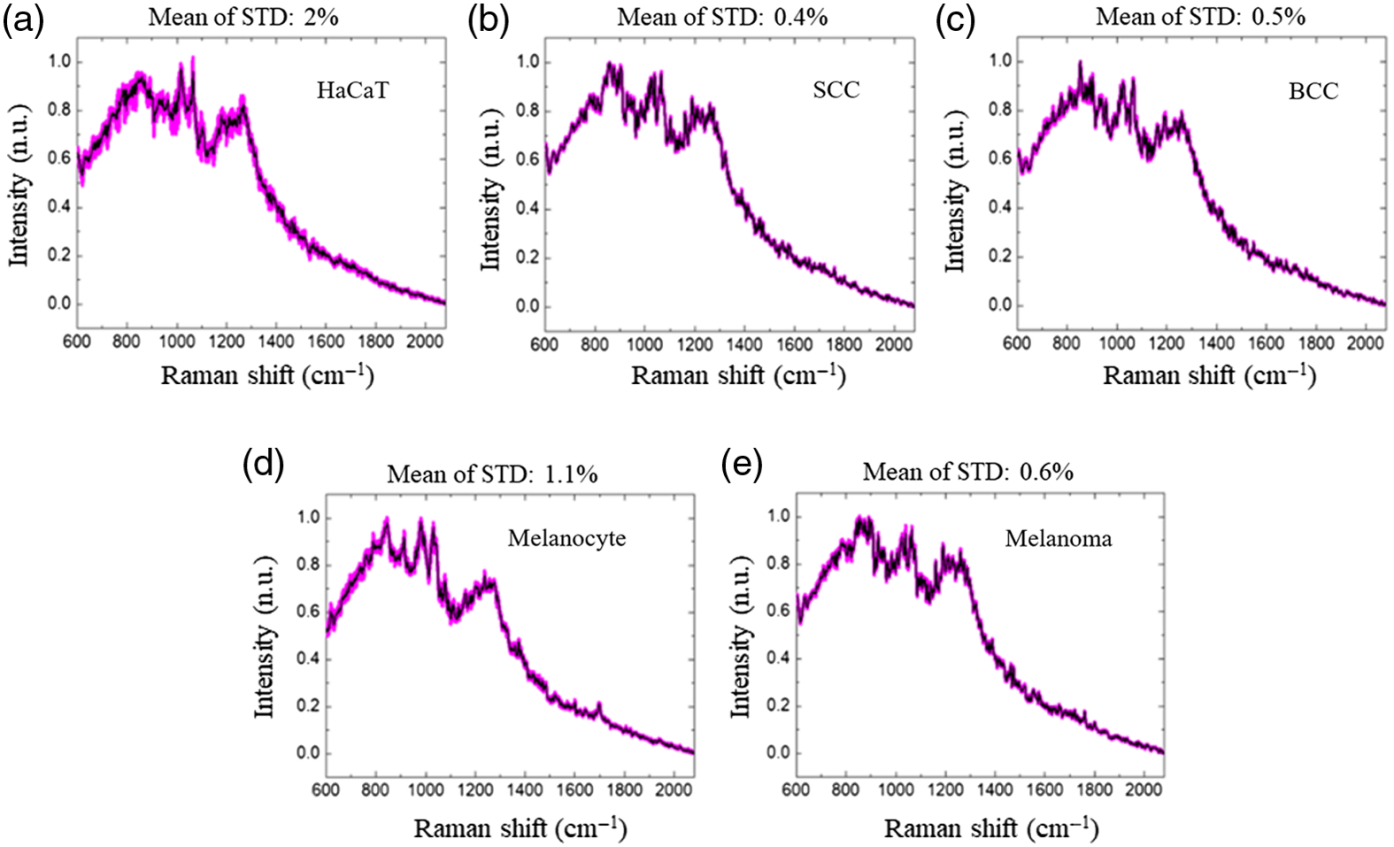
Raman spectra of (a) HaCaT (keratinocyte cell line), (b) SCC (SCC cell line), (c) BCC (BCC cell line), (d) primary melanocytes, and (e) melanoma cell line. Black lines and pink lines represent the average value and the standard deviation, respectively.

Figure 6 shows the Raman spectra obtained from the five kinds of cells where the signal from the Mirau objective is removed. The spectra are similar in a macroscopic view, and it is found that the standard deviation of the Raman spectra of normal cells (i.e., HaCaT, Melanocyte) are two to five times that of the cancer cells (i.e., SCC and BCC, and A375). The standard deviation of normal cells’ spectra is high because they may have pathological changes due to poor survival and reproduction ability.

**Fig. 7:**
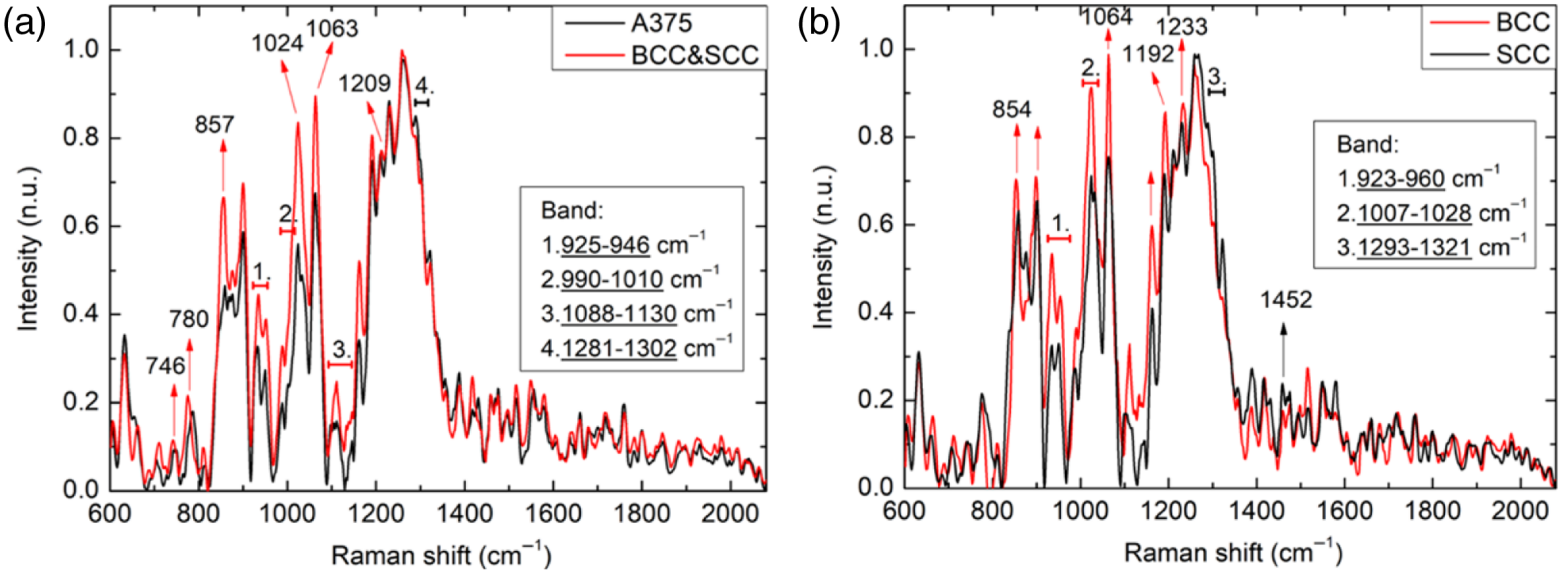
Comparison of Raman spectra. (a) Melanoma cell line (A375) versus keratinocyte-based skin cancer cell lines (BCC and SCC) and (b) keratinocyte-based skin cancer cell lines (BCC versus SCC).

After removal of the fluorescent backgrounds, it is shown in Fig. 7(a) that below the Raman shift of $1200\;{\mathrm{cm}}^{-1}$, the keratinocyte-based cell cancers (BCC and SCC), in general, have higher Raman signals, which are also depicted in the literature.^47^ Above $1220\;{\mathrm{cm}}^{-1}$, melanoma and keratinocyte-based cancer cells are comparable in signal strength. The Raman signal decreases above $1350\;{\mathrm{cm}}^{-1}$ (i.e., 872.18 nm), mainly because the detector material is germanium. Its quantum efficiency sharply declines above 875 nm, so the spectra are difficult to compare above $1350\;{\mathrm{cm}}^{-1}$. The detailed biochemical characteristics of the molecules associated with the spectral ranges are shown in Table S1 in the Supplementary Material.^47^^,^^48^ Some spectral components such as $780\;{\mathrm{cm}}^{-1}$, 925 to $946\;{\mathrm{cm}}^{-1}$, 990 to $1010\;{\mathrm{cm}}^{-1}$, 1281 to $1302\;{\mathrm{cm}}^{-1}$ have peak shifts ranging from 3 to $12\;{\mathrm{cm}}^{-1}$ as compared with those in the literature. The A375 cell line results show that nearly 80% of the spectrum calculated in 600 to $1350\;{\mathrm{cm}}^{-1}$ is comparable to the Raman spectrum from the literature.^47^^,^^48^

The Raman spectra comparison of BCC and SCC cell lines is shown in Fig. 7(b). It can be found that BCC has a higher Raman signal below $1250\;{\mathrm{cm}}^{-1}$, which is consistent with that in the literature.^47^ Above $1250\;{\mathrm{cm}}^{-1}$, the SCC Raman signal prevails from BCC.^47^ However, the spectra are difficult to compare because the quantum efficiency of the photodetector has a sharp decline beyond $1350\;{\mathrm{cm}}^{-1}$ (872.18 nm). The detailed biochemical characteristics of the molecules associated with the spectral ranges are shown in Table S2 in the Supplementary Material.^47^^–^^50^ The Raman spectra of the two cell lines have nearly 88% that can be corresponded to those in the literature between 900 and $1350\;{\mathrm{cm}}^{-1}$.^47^

### 3.3 Results of Machine Learning on OCT Image Features and Raman Spectra

**Fig. 8:**
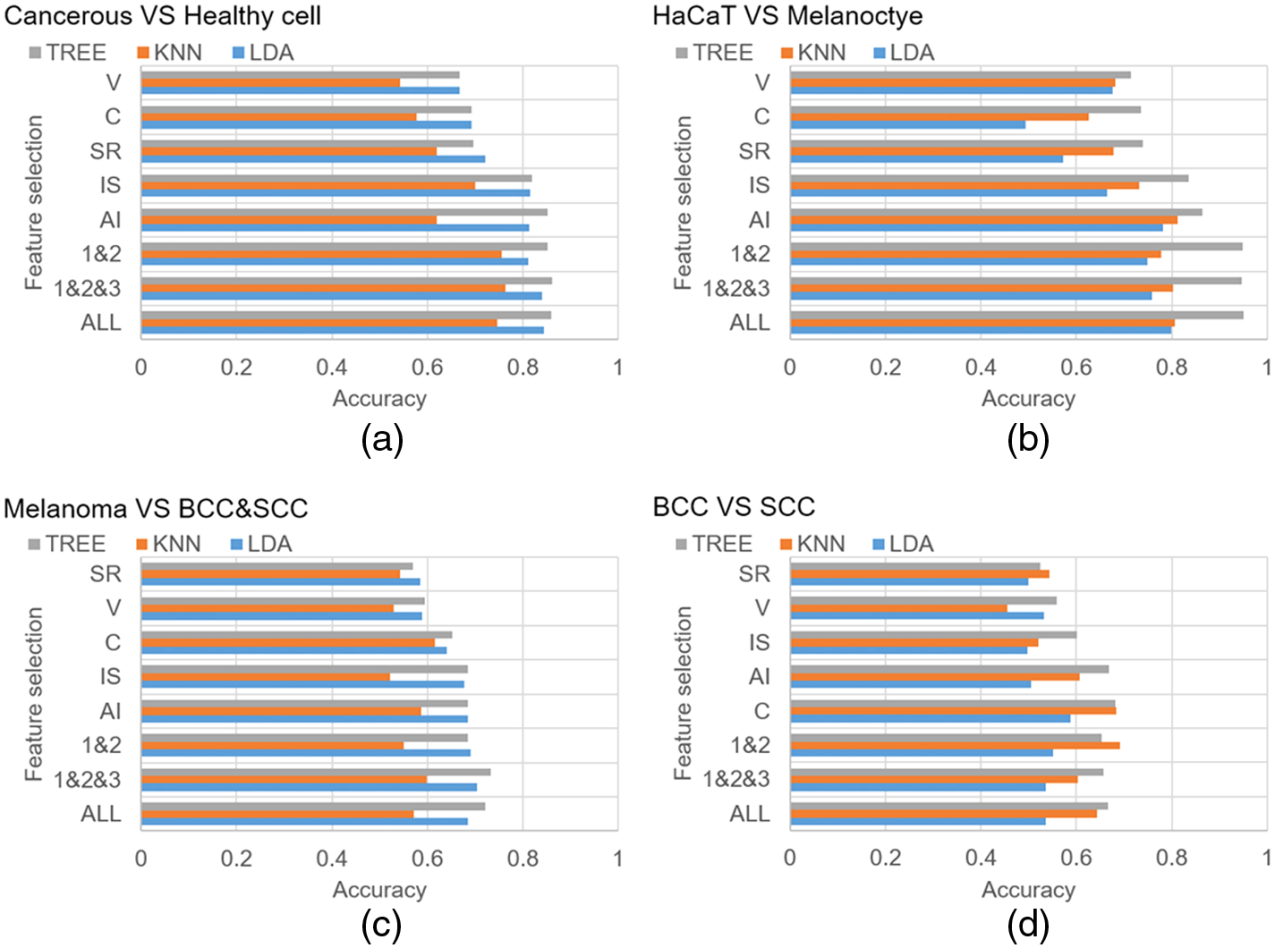
Discrimination accuracies using 3D OCT cell image features by machine learning algorithms (TREE, decision tree; KNN, K-nearest neighbors; LDA, linear discriminant analysis) on (a) cancerous versus normal cell lines, (b) keratinocyte cell line (HaCaT) versus melanocyte, (c) melanoma versus keratinocyte-based skin cancer cell lines, (d) BCC versus SCC. (V, volume; C, compactness; SR, surface roughness; IS, intensity sigma; AI, average intensity; 1&2, first two top discriminant features; 1&2&3, first three top discriminant features; ALL, all features.).

Figure 8 shows the discrimination accuracies of applying machine learning algorithms to the 3D OCT image features. The three classifiers are enhanced by the ensemble architectures. Six algorithms were attempted, i.e., boosting + LDA, bagging + LDA, subspace + LDA, subspace + KNN, boosting + TREE, and bagging + TREE. For each of the classifiers, only the best result is chosen in Fig. 8. The classifications of cancer cells with normal cells [Fig. 8(a)] and normal keratinocytes with melanocyte cells [Fig. 8(b)] both have good discrimination results. The mean intracellular intensity (MI) and the standard deviation of intensity (IS) have results with higher accuracy, and each can achieve an accuracy of more than 85%, mainly due to the slight difference in the 3D characteristics between cancer cells and normal cells. In particular, the mean intensity and standard deviation of intensity distribution may differ depending on whether or not it is a cancer cell or a normal cell. When combining the best features of these two categories, the accuracy is also quite high in the keratinocyte cell lines compared with melanocyte cells. The main reason is that the morphological differences in cell size and shape of these two kinds of cells are quite different, so the melanocytes and keratinocyte cells can be better discriminated. However, the classification ability of the OCT images is relatively low for the classification among cancer cells, only reaching nearly 70% accuracy, as shown in Figs. 8(c) and 8(d). It is speculated that although cancer cells can be clearly distinguished from normal cells, there is no significant difference in the morphological features among the cancer cells. Since there are significant chemical composition differences in cancer cells,^47^^,^^48^ RS is used as an aid. The Raman spectra show a weaker classification ability between normal cells (i.e., large standard deviation in the Raman spectra of normal cells in Fig. 6). It could be superior in the classification of cancer cells with its chemical composition specificity capability.

The full spectra (600 to $2100\;{\mathrm{cm}}^{-1}$) of the three cancer cell lines are used. Table 1 summarizes the result using the wavenumber-dependent signal intensities. All three cancer cells can be discriminated against using the Raman spectra, and the classification accuracy is near 100%. However, the time for calculating Raman spectral feature is 3 to 10 times that of the 3D OCT image feature [i.e., 2 to 5 s (OCT) versus 6 to 50 s (Raman spectra)]. The advantages and disadvantages of OCT 3D image features and Raman spectra are complementary. OCT is good at distinguishing between normal cell lines, and normal and cancer cell lines. Raman is suitable for discriminating cancer cell lines. Integrating the two can help improve the differentiation between skin cancer and normal cell lines.

**Table 1:** Discrimination accuracies using all Raman spectra by machine learning algorithms (LDA, linear discriminant analysis; KNN, K-nearest neighbors; TREE, decision tree) on (a) melanoma versus keratinocyte-based skin cancer cell lines and (b) BCC versus SCC.

	Accuracy	LDA	KNN	TREE
(a)	Melanoma versus BCC&SCC	0.859	**0.989**	0.916
(b)	BCC versus SCC	0.833	**1.000**	0.865

## 4 Conclusion

This study presents the integration of OCT with near-infrared RS. The biological sample’s stereoscopic cellular structure and molecular chemistry information are simultaneously measured. Five *in vitro* skin cells were measured and classified using machine learning algorithms. The cell features from the 3D OCT images and the full Raman spectra were fed into the ensemble machine learning algorithms for cell discrimination. The OCT scans the *in vitro* cell samples and uses image stitching to obtain a 3D tomogram of 1166.4  μm×878.4  μm×200  μm each time. A total of 283 skin cells were taken from multiple images of the OCT for analysis. In the OCT image, it can be found that the surface roughness of cancer cells is significantly higher than that of normal cells, mainly related to the intense aggressiveness of cancer cells, so there are irregular protrusion shapes on the cell surface. In addition, in terms of intensity, the cancer cells in the keratinocyte-based cells are brighter than the normal keratinocytes. The main reason is that the normal keratinocytes are more homogeneous in structure, and the surface of the cancer cells is uneven. The melanocyte-based cells show the opposite behavior. The reason is that primary melanin cells have fewer reproductive generations, which are closer to the original living conditions, and melanocytes produce melanin in large quantities when they are *in vivo*. Therefore, the melanoma cell line that has been propagated for many generations from the living body has a low melanin content, so it is lower in intensity than the primary melanin cells.

Using the Raman spectra to classify the cells, it was found that the standard deviation of normal cells is two to five times that of cancer cells, which significantly reduces the accuracy of normal cell classification. Using the 3D OCT features by the decision tree algorithm, a discrimination accuracy of 85.9% was achieved between cancerous and normal cells. Also, these 3D features can distinguish keratinocytes and melanocytes. The Raman spectra can completely classify the three kinds of cancerous cells. Integrating the cellular-resolution OCT with near-infrared RS can be an important diagnostic tool and direction for future clinical studies.

## Code, Data, and Materials Availability

Data are available upon reasonable request.
