# Supplementary material for: Integration of cellular-resolution optical coherence tomography and Raman spectroscopy for discrimination of skin cancer cells with machine learning
Source: J Biomed Opt. 2023 Sep 14;28(9):096005. doi: 10.1117/1.JBO.28.9.096005 (PMC10500347; doi:10.1117/1.JBO.28.9.096005)
Supplement: Supplementary file 1 [file JBO_028_096005_SD001.pdf]

Integration of cellular-resolution optical coherence tomography and Raman spectroscopy for discrimination of skin cancer cells with machine learning

Cian You,[a] Jui-Yun Yi,[b] Ting-Wei Hsu,[a] and Sheng-Lung Huang[a,c]

[a]National Taiwan University, Graduate Institute of Photonics and Optoelectronics, Taipei, Taiwan
[b]National Kaohsiung Normal University, Department of Electrical Engineering, Kaohsiung, Taiwan
[c]National Taiwan University, All Vista Healthcare Center, Taipei, Taiwan

Supplementary on Raman peaks of skin cancer cell lines

1. Raman peaks of melanocytic carcinoma and keratinocyte carcinoma cell lines

Listed in Table S1 are the Raman peaks corresponding to the literature,[43–45] such as 780 cm^{-1}, 925–946 cm^{-1}, 990–1010 cm^{-1}, and 1281–1302 cm^{-1} have peak differences from 3 cm^{-1} to 12 cm^{-1}. The discrepancy could be attributed to the lack of our spectrometer resolution (i.e., 6 cm^{-1}) and the low signal-to-noise ratio due to the influence of the Mirau composition. About 80% of the A375 cell lines' Raman peaks in 600–1350 cm^{-1} can find correspondence to those in the literature. As shown in Table S1, the high value of keratin-rich skin cancer cells have a Raman peak of 746 cm^{-1} from thymine in DNA, and the 780-cm^{-1} peak is from the symmetric stretching of PO_2 of nucleic acid backbone. The 857-cm^{-1} peak comprises the bending of C-C-H of protein in (855 cm^{-1}) and vibration of the C-C of collagen backbone (856 cm^{-1}). The 925–946 cm^{-1} band is composed of C-C skeletal stretching (920–943 cm^{-1}) in the collagen backbone. The 990–1010 cm^{-1} band is composed of amphetamine at 1000–1010 cm^{-1}. The 1024-cm^{-1} peak is composed of C-N stretching vibration in urea, 1063 cm^{-1} is caused by C-C asymmetric skeletal stretching in lipids. The 1088–1130 cm^{-1} band is composed of C-C stretching (1085–1098 cm^{-1}) in lipids, PO_2 symmetric stretching of the nucleic acid backbone, plus lipids' C-C symmetric stretching (1127–1130 cm^{-1}) in the acyl-backbone. The 1209-cm^{-1} peak is contributed by tyrosine and phenylalanine from 1207–1209 cm^{-1}. The high spectral content (1281–1302 cm^{-1}) of melanoma is composed of a small amount of Amide-III at 1269 cm^{-1}, C-N stretching and N-H in-plane bending plus CH_2 twisting and wagging in lipids around 1301 cm^{-1}.

Table S1 Raman shift and band assignment of melanocytic carcinoma and keratinocyte carcinoma cell lines[43]-[45]

Raman shift (cm^{-1})	Band assignment	Component
746	Thymine	DNA
786-788	PO_2 symmetric stretching	Nucleic acid backbone
855	C-C-H bending	Protein
856	C-C vibration	Collagen backbone
920-943	C-C skeletal stretching	Collagen backbone
1000-1010	Ring breathing	Phenylalanine
1024	C-N stretching vibration	Urea
1063	C-C asymmetric skeletal stretching	Lipid
1085-1098	C-C stretching	Lipid
	PO_2 symmetric stretching	Nucleic acid backbone
1127-1130	Lipids C-C symmetric stretching	Acyl backbone
1207-1209	Tyrosine and phenylalanine	
1269	Amide-III, C-N stretching, N-H in-plane bending	Collagen, elastin, and keratin
1301	C-H modes (CH_2 twisting and wagging) and CH_2/CH_3 bands	Lipid and triolein

2. Raman peaks of BCC and SCC cell lines

It can be found in Fig. 7(b) of the main manuscript that before the Raman shift of 1250 cm^{-1}, BCC has a higher Raman signal, which echoes the literature that mentioned that BCC dominates the Raman spectrum between 850–1250 cm^{-1}.[43] After 1250 cm^{-1}, it is mainly dominated by SCC.[43] Beyond 1350 cm^{-1}, the quantum efficiency of the photodiode tends to decline sharply. Only the Raman peak 1452 cm^{-1} with a large difference between the two has been found in the literature. The detailed Raman shift and band assignment of the BCC and SCC cell lines can be seen in Table S2.

About 88% of the BCC and SCC cell lines' Raman peaks in 900–1350 cm^{-1} can find correspondence to those in the literature. Before 1400 cm^{-1}, except for the peak positions of 898, 1158, and 1191 cm^{-1}, other peaks marked with red arrows in Fig. 7(b) can all find correspondence in the literature. However, the biomedical components corresponding to these three peak positions are not assigned in the literature, so they are not listed in Table S2. These peak positions will also be put into the classification algorithm and processed together.

Table S2 Raman shift and band assignment of BCC and SCC cell lines[43]-[46]

Raman shift (cm^{-1})	Band assignment	Component
855	C-C-H bending	Protein
856	C-C vibration	Collagen backbone
920-943	C-C skeletal stretching	Collagen backbone
1000-1010	Ring breathing	Phenylalanine
1024	C-N stretching vibration	Urea
1063	C-C asymmetric skeletal stretching	Lipid
1230-1240	Amide-III (β fold)	Protein
1288-1304	Bending vibration of CH_2 and CH_2CH_3	Lipid
1320-1340	CH_2CH_3 stretching and bending	Protein and nucleic acid
	C-H bending	Unsaturated fatty acid
1440-1460	CH_2 bending	Lipid and protein
